# The Association Between Health, Social Engagement, and Psychological Well-Being Among Older Adults in China

**DOI:** 10.1093/geroni/igaa057.315

**Published:** 2020-12-16

**Authors:** Yuekang Li

**Affiliations:** Brown School, Washington University in St. Louis, St. Louis, Missouri, United States

## Abstract

Psychological health and health-related quality of life of older adults have been long minimized by caregivers, service providers and the society in developing countries, such as China. According to the Stress Process Model, the stress of physical disadvantages influences mental health outcomes directly and indirectly. However, being socially engaged has the potential to reduce disease burden and improve psychological wellbeing of older adults. The purpose of this study is to examine the role played by social engagement in the pathway through which physical health predicts mental health. Using the WHO Study on Global AGEing and Adult Health China wave 2010, 6,276 individuals ages 60 years and older were included for analyses. Structural equation modeling was used to construct a directional path leading from the functioning and chronic disease, impacting the social engagement, in turn impacting the psychological wellbeing. All variables in this model are latent constructs. Functioning and chronic diseases in later life were associated with social engagement and psychological wellbeing, and the link between social engagement and psychological wellbeing was also significant. The effect of function was greater than that of chronic diseases. Though the significant indirect effect of physical health on psychological wellbeing was not supported in this study, this study suggests the multiple roles of social engagement as coping resources in the stress process of physical impairment and illness of older adults. This present study also adds to the existing literature by exploring how SEM methods can be applied to studies of social engagement.

